# Dosing optimization of CCR4 immunotoxin for improved depletion of CCR4^+^ Treg in nonhuman primates

**DOI:** 10.1002/1878-0261.12331

**Published:** 2018-07-03

**Authors:** Zhaohui Wang, Nathan J. Louras, Alexandre G. Lellouch, Shannon G. Pratts, Huiping Zhang, Haoyu Wang, Christene A. Huang, Curtis L. Cetrulo, Joren C. Madsen, David H. Sachs, Zhirui Wang

**Affiliations:** ^1^ Center for Transplantation Sciences Massachusetts General Hospital and Harvard Medical School Boston MA USA; ^2^ Division of Cardiac Surgery Massachusetts General Hospital and Harvard Medical School Boston MA USA; ^3^ TBRC Laboratories Center for Transplantation Sciences Massachusetts General Hospital and Harvard Medical School Boston MA USA

**Keywords:** CCR4, diphtheria toxin, immunotoxin, Treg

## Abstract

Recently, we have developed a diphtheria toxin‐based recombinant anti‐human CCR4 immunotoxin for targeting CCR4^+^ tumors and Tregs. In this study, we further optimized the dosing schedule for improved CCR4^+^ Treg depletion. We have demonstrated that up to a 90% depletion was achieved and the depletion extended to approximately 2 weeks in the peripheral blood and more than 48 days in the lymph node at 25 μg·kg^−1^, BID for 8 consecutive days in cynomolgus monkeys. Expansion was observed including monocytes and NK cells. Antibody against the CCR4 immunotoxin was detected after approximately 2 weeks, affecting further depletion efficacy for multiple course treatment.

**Abbreviations**
AAALAC: Association for Assessment and Accreditation of Laboratory Animal Care
ABTS: 2,2′‐Azino‐bis(3‐ethylbenzthiazoline‐6‐sulfonic acid)
ADCC: antibody‐dependent cellular cytotoxicity
ADCP: antibody‐dependent cellular phagocytosis
APC: allophycocyanin
BD: Becton Dickinson
BID: bis in die
BL‐2: biosafety level 2
CCR4: CC (C‐C motif) chemokine receptor 4
CDC: complement‐dependent cytotoxicity
CTLA‐4: cytotoxic T‐lymphocyte‐associated protein 4
DT390: first 390 amino acids of the diphtheria toxin
ELISA: enzyme‐linked immunosorbent assay
FDA: food and drug administration
FITC: fluorescein isothiocyanate
Foxp3: forkhead box P3
GITR: glucocorticoid‐induced tumor necrosis factor receptor
HLA‐DR: human leukocyte antigen‐antigen D related
HRP: horseradish peroxidase
IACUC: Institutional Animal Care and Use Committee
IgG: immunoglobulin G
IJV: internal jugular vein
IV: intravenous
LAG3: lymphocyte‐activation protein 3
mAbs: monoclonal antibodies
MGH: Massachusetts General Hospital
NK cells: natural killer cells
NOD/SCID: nonobese diabetic/severe combined immunodeficiency
*NSG*: NOD/SCID IL‐2 receptor γ^−/−^
OX40: tumor necrosis factor receptor superfamily member 4
PBMC: peripheral blood mononuclear cell
PBS: phosphate‐buffered saline
PBST: phosphate‐buffered saline with Tween‐20
PD‐1: programmed cell death protein 1
PE: phycoerythrin
PerCP: peridinin chlorophyll protein
SDS: sodium dodecyl sulfate
SEM: standard error of the mean
Tregs: regulatory T cells
VLS: vascular leak syndrome

## 1 Introduction

Regulatory T cells (Tregs) are a highly immune‐suppressive subset of CD4^+^ T cells and specifically express transcription factor Foxp3. Tregs suppress abnormal immune response against self‐antigen and also suppress antitumor immune response. Treg depletion or induction is a new therapeutic strategy for treating various diseases including autoimmune diseases, cancers, and transplantation tolerance induction (Hall, 2016; Liu *et al*., 2016; Lu *et al*., 2017; Romano *et al*., 2017; Tanaka and Sakaguchi, 2017). Chemokine (C‐C motif) receptor 4 (CCR4) is recognized as an important effector Treg target (Sugiyama *et al*., 2013). Scientists are exploring different approaches to develop effective and specific CCR4^+^ effector Treg depletion agents. Recently, we have developed a diphtheria toxin‐based recombinant anti‐human CCR4 immunotoxin using a unique diphtheria toxin‐resistant yeast *Pichia Pastoris* expression system (Wang *et al*., 2015). The *in vivo* efficacy for targeting human CCR4^+^ tumors was characterized using the human CCR4^+^ tumor‐bearing immunodeficient *NSG* mouse model (Wang *et al*., 2015). The *in vivo* efficacy for depleting CCR4^+^ Tregs was characterized using naive cynomolgus monkeys (Wang *et al*., 2016). Monkey CCR4^+^Foxp3^+^ Tregs were effectively depleted in both peripheral blood and lymph nodes (Wang *et al*., 2016). However, the depletion only lasted for approximately 1 week in the peripheral blood, which may not be sufficient for some applications. Clinically, the animals were healthy and exhibited no apparent side effects throughout the entirety of the study. The data indicated that there was still room for further dose escalation and increased treatment duration, which may further enhance CCR4^+^ Treg depletion. In this study, we further optimized the dosing schedule for better efficacy and improved duration of monkey CCR4^+^ Treg depletion.

## 2 Materials and methods

### 2.1 Antibodies and immunotoxin

All antibodies used in this study are listed in Table 1. The single‐chain foldback diabody anti‐human CCR4 immunotoxin was developed and produced in our laboratory using a unique diphtheria toxin‐resistant yeast *Pichia Pastoris* expression system (Wang *et al*., 2015).

**Table 1:** Antibodies used in this study

Antibody name	Clone#	Source	Cat#
FITC‐Mouse Anti‐Human CD14	M5E2	BioLegend (San Diego, CA, USA)	301804
FITC‐Mouse Anti‐Human CD16	3G8	BioLegend	302006
FITC‐Mouse Anti‐Human CD196 (CCR6)	G034E3	BioLegend	353412
FITC‐Mouse Anti‐Human CD20	2H7	BioLegend	302304
PE‐Mouse Anti‐Human CD194 (CCR4)	L291H4	BioLegend	359412
PE‐Mouse Anti‐Human NKp80	5D12	BioLegend	346706
PE‐Mouse Anti‐Human CD20	2H7	BioLegend	302306
PerCP‐Mouse Anti‐Human CD16	3G8	BioLegend	302030
PerCP‐Mouse Anti‐Human HLA‐DR	L243	BioLegend	307628
APC‐ Mouse Anti‐Human CD11c	3.9	BioLegend	301614
APC‐ Hamster Anti‐Human CCR10	6588‐5	BioLegend	341506
APC‐Mouse Anti‐Human HLA‐DR	L243	BioLegend	307610
Alexa Fluor‐Mouse Anti‐Human Foxp3	206D	BioLegend	312114
PE‐Mouse IgG1, κ Isotype Ctrl	MOPC‐21	BioLegend	400112
Alexa Fluor^®^ 647 Mouse IgG1, κ Isotype	MOPC‐21	BioLegend	400130
FITC‐Mouse Anti‐Human CD3ε	SP34	BD Bioscience (San Jose, CA, USA)	556611
PE‐Mouse Anti‐Human CD123	7G3	BD Bioscience	554529
PerCP‐Mouse Anti‐Human CD4	L200	BD Bioscience	550631
APC‐Mouse Anti‐Human CD8	RPA‐T8	BD Bioscience	555369
FITC‐Mouse Anti‐Human CD45RA	5H9	BD Bioscience	556626

### 2.2 *In vivo* CCR4^+^ Treg depletion study in nonhuman primates

Four male *cynomolgus* monkeys (M10916: 7.5 kg, M11216: 8.1 kg, M11016: 7.7 kg, M11116: 7.5 kg) were purchased from Charles River (Wilmington, MA, USA) and housed at the Massachusetts General Hospital (MGH) nonhuman primate facility. All animal care procedures and experiments were performed in accordance with the guidelines set out by the Principles of Laboratory Animal Care and Guide for the Care and Use of Laboratory Animals. All diagnostic, experimental, and euthanasia procedures were approved by the Massachusetts General Hospital Institutional Animal Care and Use Committee (IACUC). MGH is an Association for Assessment and Accreditation of Laboratory Animal Care (AAALAC) accredited institute. The CCR4 immunotoxin was administered as an intravenous (IV) bolus via a central venous catheter (internal jugular vein). ~3 mL of saline was injected before and after the immunotoxin injection. Blood from each animal was collected from the internal jugular vein line daily during the immunotoxin treatment period and twice weekly thereafter. ~3 mL of saline was flushed through the central line after the blood collection to ensure complete infusion of the immunotoxin. Maintenance of the central line included a 5 mL bolus of heparinized saline (Excelsior Medical, LLC, Neptune City, NJ, USA) following each blood collection or/and immunotoxin injection to prevent catheter occlusion. In addition, the central line was continuously infused with 0.9% normal saline at a rate of 10–15 mL·h^−1^ and flushed once daily with 5 mL bolus of heparinized saline to ensure patency.

The animals were closely monitored twice daily during the immunotoxin treatment and once daily after the immunotoxin treatment for any adverse effects. Complete blood counts (daily during the immunotoxin treatment and twice weekly thereafter), weekly serum chemistries, and weekly body weight measurement were performed to monitor for any adverse events. Prior to placement of the central venous catheters, all animals underwent jacket and tether training per veterinary guidelines for 1 week each. The depletion in the peripheral blood was monitored daily during the immunotoxin treatment and twice weekly thereafter by flow cytometry analysis as described (Wang *et al*., 2016). Lymph node biopsies were performed prior to and after each course of the CCR4 immunotoxin treatment for each experimental animal. The depletion in the lymph nodes was analyzed by flow cytometry as described (Wang *et al*., 2016).

**Figure 1:**
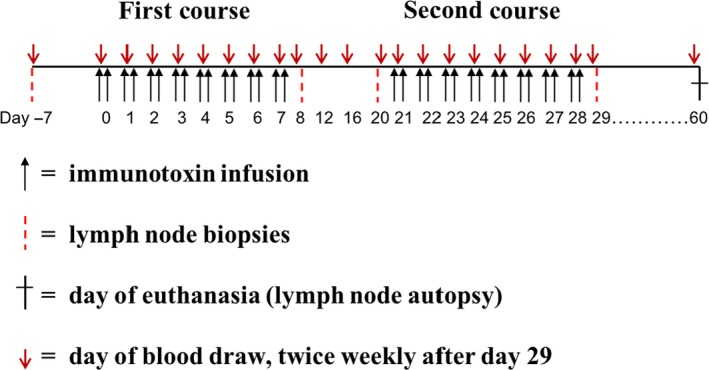
Schematic diagram of the designed dosing schedule.

Our originally designed dosing schedules (Fig. 1) included possible treatments with: (a) 25 μg·kg^−1^, (b) 50 μg·kg^−1^, or (c) 75 μg·kg^−1^, BID for eight consecutive days within total 21 days as a first round, with a second round of infusion at day 21. There would be a 13‐day break between the two rounds. The intention to inject the immunotoxin for 8 consecutive days was to prolong the depletion duration. The purpose of the second round of injections was to explore whether antibody formation against the CCR4 immunotoxin might limit the depletion efficacy of subsequent treatments. To minimize animal usage for this study, we started with the middle‐dosing schedule of 50 μg·kg^−1^, with the intention to increase the dosing to 75 μg·kg^−1^ for the next group of animals should the animals tolerated the initial dosing. If the animals were unable to tolerate the dosing, we planned to decrease the dosing to 25 μg·kg^−1^.

### 2.3 Monkey central venous catheter insertion

Animals were sedated with 20 mg·kg^−1^ ketamine intramuscularly, followed by 0.05 mg·kg^−1^ atropine intramuscularly. Animals were placed in a supine position and shaved for postoperative jacket placement. Endotracheal intubation was performed, and general anesthesia was introduced using 1–3% isoflurane and oxygen. Left neck was exposed, and a left paramedian longitudinal incision was made. The left internal jugular vein (IJV) was identified and dissected circumferentially. Next, a skin tunnel was placed from the neck incision to the recipient's midline of the back between the inferior scapulae and a single lumen catheter was pulled through the skin tunnel. The IJV was then tied distally with a 2‐0 silk tie. A venotomy was made proximally, and the IJV was cannulated. The line was inserted approximately 10 cm, situating the catheter tip inside the right atrium, approximately. The line was then secured within the vein using a 2‐0 silk tie. Blood flow and infusion with 100 U·mL^−1^ heparin sulfate in 0.9% normal saline through the line was then checked to ensure proper function.

### 2.4 ELISA analysis

ELISA analysis was performed as described (Woo *et al*., 2008, 2010). Briefly, 96‐well plate was coated with 100 μL per well of the foldback diabody anti‐human CCR4 immunotoxin at 5 μg·mL^−1^ in 1x PBS pH 7.4 for 2 h at room temperature with shaking at 70 rpm. The plate was then washed three times with PBST (1x PBS pH 7.4 + 0.1% Tween‐20) with shaking at 70 rpm for 5 min. The wells were blocked with 125 μL of 3% gelatin in 1x PBS pH7.4 for 1 h at room temperature with shaking at 70 rpm and then warmed in 37 °C incubator for ~30 min to melt the gelatin. The gelatin was then flicked out, and the plate was washed three times with PBST. The coated and blocked plate could then be used immediately or stored at −20 °C. The coated and blocked plate was washed three times with 1x PBST. The serum samples were diluted to 1 : 100 and 1 : 500 in 1x PBS pH 7.4 + 1% BSA. 100 μL of the diluted serum samples including diluted positive serum control was added to appropriate wells. A secondary antibody only control was included by adding 100 μL of 1x PBS pH 7.4. The plate was incubated at room temperature with shaking at 70 rpm for 1 h and washed three times with 1x PBST. 100 μL of the rabbit anti‐human IgG‐HRP (1 : 4000 dilution in 1x PBS pH 7.4) was added and incubated for 1 h at room temperature with shaking at 70 rpm. The plate was washed three times with 1x PBST. 100 μL of ABTS [2,2′‐Azino‐bis(3‐ethylbenzthiazoline‐6‐sulfonic acid)] (Southern Biotech, cat# 0404‐01, Birmingham, AL, USA) was added and incubated for 10 min at room temperature in the dark with shaking at 70 rpm. Fifty microliter of 1% SDS was added to stop the reaction. The absorbance value at 405 nm was collected using ELISA plate reader (Perkin Elmer Wallac 1420 Victor^2^ Multilabel Counter, Waltham, MA, USA).

### 2.5 Flow cytometry

All the monkey tissues were operated under BL‐2 rules. The cell surface and intracellular staining were performed as previously described (Wang *et al*., 2016). Flow cytometry data were collected suing a BD FACSCalibur (Becton Dickinson, California) and analyzed with FlowJo analysis software (FlowJo, LLC, Ashland, OR, USA).

## 3 Results

To avoid frequent sedation, jacket and tether training was performed and an internal jugular vein line was inserted in each of the experimental animals for frequent blood draw and immunotoxin injection. To keep the line functional, 0.9% normal saline was continually infused at a rate of 10–15 mL·h^−1^ via an infusion pump and flushed daily with a ~5 mL bolus of heparinized saline.

Peripheral blood Treg levels were measured 3 times prior to the administration of the immunotoxin to ensure accurate baseline data. Flow cytometry of the peripheral blood was performed daily during each treatment cycle and twice weekly thereafter to monitor the depletion by the immunotoxin of peripheral blood cell populations, including T cells, B cells, NK cells, and monocytes. A combination of stains for CD4, CCR4, CD45RA, and Foxp3 were used to identify Treg populations.

**Figure 2:**
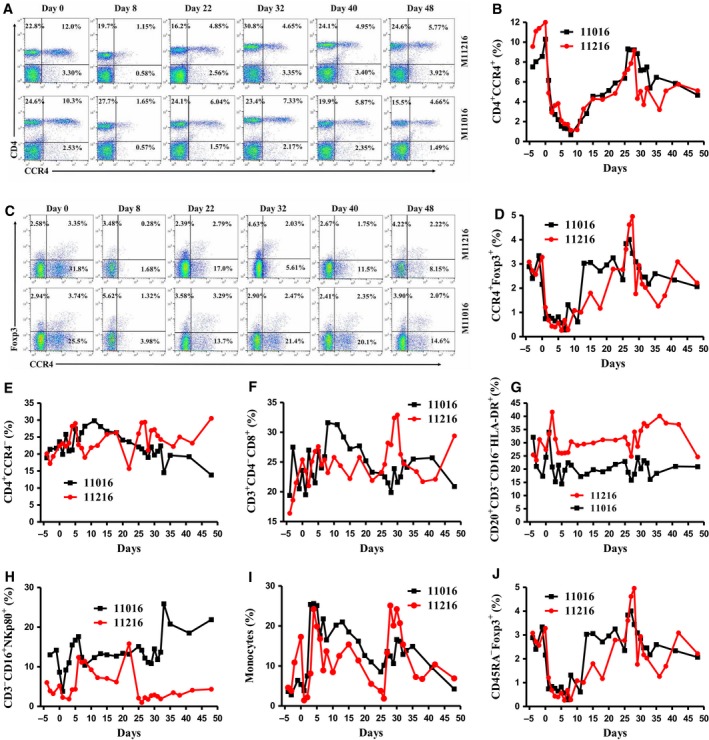
Monkey CCR4^+^ Treg depletion for animals M11016 and 11216 in the peripheral blood using the CCR4 immunotoxin. (A) Representative flow cytometry analysis of the CCR4^+^ cell depletion in the peripheral blood using the antibodies against human CD4 and CCR4. (B) CCR4^+^ cell depletion in the peripheral blood was monitored by flow cytometry using the antibodies against human CD4 and CCR4 (CD4^+^ CCR4^+^). (C) Representative flow cytometry analysis of the CCR4^+^Foxp3^+^ Treg depletion in the peripheral blood using the antibodies against human CCR4 and Foxp3 (CCR4^+^Foxp3^+^ among the gated CD4^+^ cells). (D) CCR4^+^ Treg depletion in the peripheral blood was monitored by flow cytometry using the antibodies against human CCR4 and Foxp3 (CCR4^+^Foxp3^+^ among the gated CD4^+^ cells). (E) The other CD4^+^ cells in the peripheral blood were monitored by flow cytometry using antibodies against human CD4 and CCR4 (CD4^+^ CCR4^−^). (F) The CD8^+^ T cells in the peripheral blood were monitored by flow cytometry using the antibodies against human CD3, CD4, and CD8 (CD3^+^ CD4^−^ CD8^+^). (G) The B cells in the peripheral blood were monitored by flow cytometry using antibodies against human CD20, CD3, CD16, and HLA‐DR (CD20^+^ CD3^−^ CD16^−^ HLA‐DR ^+^). (H) The NK cells in the peripheral blood were monitored using antibodies against human CD3, CD16, and NKp80 (CD3^−^ CD16^+^ NKp80^+^). (I) Monocytes in the peripheral blood were monitored by flow cytometry using antibodies against human CD14, CD16, and CD11b (CD14^+^ CD11b^+^ or CD14^+^ CD16^+^, PBMC gating). (J) The effector Tregs in the peripheral blood were monitored by flow cytometry using antibodies against CD45RA and Foxp3 (CD45RA ^‐^Foxp3^+^ among the gated CD4^+^ cells).

**Figure 3:**
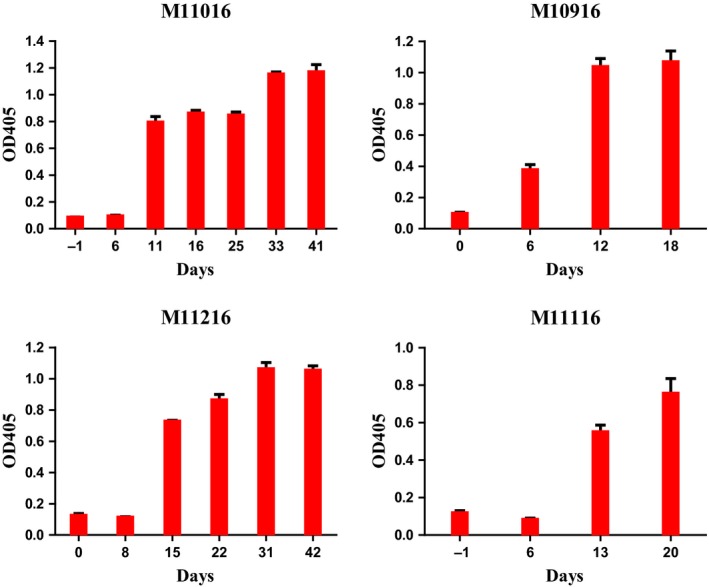
ELISA analysis of the antibody formation against the CCR4 immunotoxin for all experimental animals M11016, M11216, M10916, and M11116. Data are representative of three different individual experiments. Three repeats were included for each serum sample of individual experiment. Error bars are SEM.

For group #1 animals (M10916 and M11216) (Table 2), the CCR4 immunotoxin was injected at 50 μg·kg^−1^, BID for only 4 days due to the observed adverse effects mainly lack of appetite. For this reason, the dosing was adjusted to 25 μg·kg^−1^, BID for the remaining 4 days of the first course for animal M11216. The experiment was terminated following the 4‐day treatment for animal M10916 due to intolerance of the jacket. The depletion of CCR4^+^ cells and CCR4^+^Foxp3^+^ Tregs was prolonged from approximately 1 week (Wang *et al*., 2016) to approximately 2 weeks with similar depletion depth for the first course treatment (Fig. 2A–D). Only the flow cytometry data of animals M11216 (group #1) and M11016 (group #2 discussed later) were presented, to avoid crowded figures (other data were presented in Figs S1–S3). The results demonstrated that the prolonged immunotoxin administration extended the depletion duration. Following the 13‐day break between rounds, the second round of treatment was performed with animal M11216 at 25 μg·kg^−1^, BID for eight consecutive days. During the second round of treatment, nearly no depletion was observed for CCR4^+^ cells or CCR4^+^Foxp3^+^ Tregs in the peripheral blood (Fig. 2B,D). ELISA analysis demonstrated that antibody formation against the CCR4 immunotoxin occurred at around 2 weeks (Fig. 3). The depletion was negatively correlated with this antibody formation. Thus, the data demonstrated that the efficacy of the immunotoxin's depletion was affected by the presence of the antibody.

**Table 2:** Dosing optimization study summary

Animal#	Group#	1st course			2nd course			
		Dose (μg·kg^−1^)	Days	Depletion	Antibody Formation^a^	Dose (μg·kg^−1^)	Days	Depletion
M10916	1	50	4	**++++**	Yes			
M11216	1	50	4	**++++**	Yes	25	8	–
		25	4					
M11016	2	25	8	**++++**	Yes	25	8	–
M11116	2	25	8	**++++**	Yes	25	3	–

**++++** more than 80% of CCR4^+^Foxp3^+^ Treg depletion; **–** almost no depletion of CCR4^+^Foxp3^+^ Treg.

^a^Antibody formation against the CCR4 immunotoxin at around 2 weeks.

Given that 50 μg·kg^−1^ was too high to be tolerated for group #1 animals, the CCR4 immunotoxin was administered at an initial dose of 25 μg·kg^−1^, BID for 8 consecutive days for group #2 animals (M11016 and M11116) (Table 2). As expected, the depletion profile for both animals was similar to that of group #1 animal, M11216, for the first course of treatment in the peripheral blood (Fig. 2A–D). Following the 13‐day intermission, the entire second course treatment was completed with animal M11016 at 25 μg·kg^−1^, BID for 8 consecutive days. The depletion profile for the second course was similar to that of group #1 animal M11216 (Fig. 2A–D). Unfortunately, the experiment was terminated for M11116 after only 3 days of the second course treatment at 25 μg·kg^−1^, BID due to complications following one of the routine procedures (data not shown). The main complication was a bleeding event (technical reason, not due to the immunotoxin treatment) following one of the scheduled lymph node biopsies. No depletion of the CCR4^+^Foxp3^+^ Tregs was observed in the peripheral blood following 3 days of the second course. ELISA analysis confirmed antibody formation at approximately 2 weeks following the first treatment course for both animals M11016 and M11116 (Fig. 3).

The specificity of the CCR4^+^ cell deletion and effect on other cell lineages was monitored by flow cytometry. Antibodies were used to detect the populations of nonhuman primate (NHP) T cells, B cells, NK cells, and monocytes (other CD4^+^ T cells: CD4^+^CCR4^−^; CD8^+^ T cells: CD8^+^CD3^+^CD4^−^; B cells: CD20^+^CD3^−^CD16^−^HLA‐DR^+^; NK cells: CD3^−^CD16^+^NKp80^+^; Monocytes: CD14^+^CD11b^+^ or CD14^+^CD16^+^; effector Tregs: CD45RA^‐^Foxp3^+^). No off‐target depletion was observed among CD4^+^CCR4^−^ T cells (Fig. 2E), CD8^+^ T cells (Fig. 2F), or B cells (Fig. 2G) in the peripheral blood. However, expansion of NK cells (Fig. 2H) and monocytes (Fig. 2I) was observed. CD45RA^‐^Foxp3^+^ effector Treg depletion was observed with the first course treatment (Fig. 2J).

**Figure 4:**
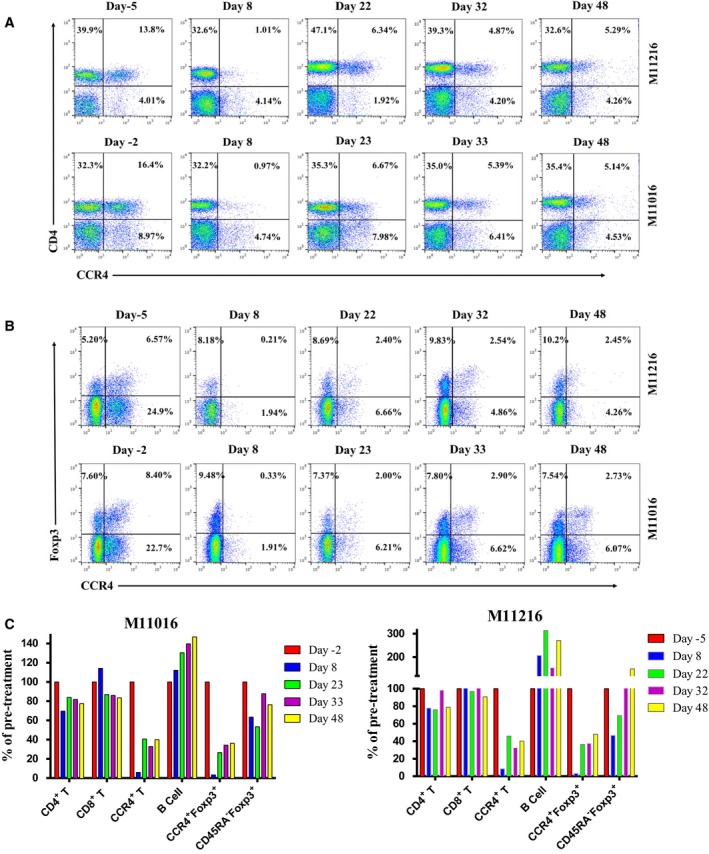
Monkey CCR4^+^ Treg depletion for animals M11016 and 11216 in the lymph node using the CCR4 immunotoxin. The lymph node biopsies were performed before and after each course of the immunotoxin treatment as well as in the end of the study. (A) Flow cytometry analysis of the lymph node biopsy samples using antibodies against human CD4 and CCR4. (B) Flow cytometry analysis of the lymph node biopsy samples using antibodies against human CCR4 and Foxp3 (CCR4^+^Foxp3^+^ among the gated CD4^+^ cells). (C) Lymph node CCR4^+^ Treg depletion was monitored by flow cytometry (CCR4^+^ cells: CD4^+^ CCR4^+^, CCR4^+^ Tregs: CCR4^+^Foxp3^+^ among the gated CD4^+^ cells, effector Tregs: CD45RA ^‐^Foxp3^+^ among the gated CD4^+^ cells). Other cell populations in the lymph node were also monitored by flow cytometry (other CD4^+^ cells: CD4^+^ CCR4^−^; CD8^+^ cells: CD8^+^ CD3^+^ CD4^−^; B cells: CD20^+^ CD3^−^ CD16^−^ HLA‐DR ^+^).

Lymph node biopsies were performed before and after each round of the immunotoxin treatment. However, due to the poor body condition, no lymph node biopsy was performed prior to the second treatment course for M11116. The percentage of Tregs in each of lymph node biopsies was analyzed by flow cytometry (CD4, CD45RA, CCR4, and Foxp3). As shown in Fig. 4A–C, both the CCR4^+^ cells and the CCR4^+^Foxp3^+^ Tregs from the lymph nodes were successfully depleted following the first treatment course. Prior to and following the second round of treatment, the depletion rebounded back to ~40% of pretreatment level. Even at the end of entire study (day 48), the rebound level was still at ~40%. No depletion was observed on CD8^+^ T cells, other CD4^+^ T cells, or B cells from the lymph node (Fig. 4C). B‐cell expansion was observed in the lymph node following the 8‐day treatment with animals M11016, M11116, and M11216 (Figs 4C and S3C).

Clinically, animals (M11016 and M11216) were healthy throughout the entire study. All the serum chemistry analysis results were within the normal range or minimally affected (data not shown). The main adverse effect observed was a loss of appetite during the immunotoxin treatment. This adverse effect may have in part been attributed to the stress caused by wearing the jacket and tether. Early termination for M10916 and M11116 was not related to the immunotoxin treatment. Transient lymphopenia was also observed (Fig. S2K) as reported previously (Wang *et al*., 2016).

## 4 Discussion

Treg‐targeted antitumor immunotherapy has attracted increasing attention. Molecules expressed relatively specifically on Tregs are good targets including CCR4, CD25, CTLA‐4, GITR, PD‐1, OX40, and LAG3. Monoclonal antibodies such as mogamulizumab have demonstrated promising Treg depletion efficacy (Tanaka and Sakaguchi, 2017). Unlike therapeutic monoclonal antibodies (mAbs), the therapeutic effect of CCR4 immunotoxin does not rely on accessory cells from the innate immune system to initiate antibody‐dependent cellular cytotoxicity (ADCC), complement‐dependent cytotoxicity (CDC), or antibody‐dependent cellular phagocytosis (ADCP). CCR4 immunotoxin is therefore expected to avoid resistance mechanisms attributed to poor accessory cell function in immunocompromised and heavily pretreated cancer patients, as commonly occur with mAb therapy (Vaughan *et al*., 2015; Vela *et al*., 2015). In addition, therapeutic antibodies function very poorly on some particular tissues such as bone marrow and skin (personal communication). CCR4 immunotoxin will be an ideal drug candidate for those particular applications. *In vivo* half‐life of the immunotoxin is very short (~60 min). Transient CCR4^+^ effector Treg depletion will be advantages to avoid the risk of autoimmune diseases. We believe that the CCR4 immunotoxin will be one of the promising Treg‐targeted immunotherapy drug candidate for combined cancer treatment.

B‐cell expansion (Fig. 4C) was observed in the lymph nodes due to the CCR4 immunotoxin treatment. We speculate that the B‐cell expansion was also a possible functional indication of the Treg depletion (Mayer *et al*., 2014). Monkey CCR4^+^ Tregs may also directly suppress the maturation of B cells similar to the effect of CCR4^+^ Tregs on suppression of dendritic cell maturation (Bayry *et al*., 2008).

As in the treatment with Ontak^®^ (Eisai Co., Ltd., Tokyo, Japan), we expected that the CCR4 immunotoxin would remain effective throughout the second course of treatment, given that both medications have identical DT390 portion (truncated diphtheria toxin, first 390 amino acids of the diphtheria toxin). Ontak^®^ has been approved by FDA for repeated dosing (9 or 18 μg·kg^−1^·day^−1^ by intravenous infusion over 30–60 min for five consecutive days every 21 days for eight cycles). Even though the antibody was detected against Ontak^®^ from the treated patients, the formed antibodies did not affect the Ontak^®^ efficacy for the subsequent multiple course treatment (Kaminetzky and Hymes, 2008). The exact mechanism remains unclear. In contrast, our data demonstrated that the CCR4 immunotoxin efficacy during the second round of treatment was significantly affected by the antibody formation, at least in the peripheral blood. Lymph nodes from both M11016 and M11216 (Fig. 4C) showed prolonged depletion of CCR4^+^ Tregs following 8‐day CCR4 immunotoxin treatment with CCR4^+^ Treg levels returning to only ~40% of pretreatment by day 48. In contrast, the level of CCR4^+^ Tregs returned to baseline by day 22 in M10916 (Fig. S3C), likely due to the short treatment course (4 days). Prolonged depletion of CCR4^+^ Treg in the lymph nodes may prove to be beneficial in cancer treatment. More studies need to be performed as these results may be due to difference in the immunotoxin efficacy between naive animals and the immune compromised patients with cancer. In cases where multiple treatment courses of CCR4 immunotoxin are needed, it may be necessary to add immunosuppressive agents such as cyclosporine.

Ontak^®^ has a black box warning for vascular leak syndrome (VLS). However, we did not observe any peripheral edema or pulmonary edema during the entire study. At necropsy, we did not observe any tissue edema or perivascular cuffing. One possible explanation is that the CCR4 immunotoxin was expressed using an advanced diphtheria toxin‐resistant yeast *Pichia pastoris* expression system and the final protein product quality is better than *Escherichia coli* expressed and refolded Ontak^®^ final protein product. Second possible explanation is the difference between human IL‐2 portion versus the anti‐human CCR4 scFv portion as human IL‐2 alone can cause VLS too (Baluna and Vitetta, 1997).

## 5 Conclusion

A novel diphtheria toxin‐based recombinant anti‐human CCR4 immunotoxin capable of depleting human CCR4^+^ tumors and Tregs was evaluated for efficacy *in vivo* in cynomolgus monkeys. In this study, a modified dosing strategy was tested for improved depletion of CCR4^+^ Tregs. We demonstrated up to 90% depletion for approximately 14 days in the peripheral blood and for more than 48 days in the lymph node using a modified dosing strategy of 25 μg·kg^−1^ BID for eight consecutive days.

## Author contributions

Zhaohui Wang primarily performed the experiments, data analysis, and manuscript preparation. AGL and NJL performed the surgeries. NJL participated in the manuscript writing. SGP, HZ, and HW participated in the experiments. CAH, DHS, JCM, and CLC participated in the project design, data analysis, and manuscript writing. Zhirui Wang primarily designed the project, analyzed the data, and wrote the manuscript.

## Supporting information


**Fig. S1.** Monkey CCR4^+^ Treg depletion (absolute count curves) for M11016 and M11216 in the peripheral blood using the CCR4 immunotoxin.
**Fig. S2.** Monkey CCR4^+^ Treg depletion for M10916 and M11116 in the peripheral blood using the CCR4 immunotoxin.
**Fig. S3.** Monkey CCR4^+^ Treg depletion for M10916 and M11116 in the lymph node using the CCR4 immunotoxin.
