# Supplementary material for: Dosing optimization of CCR4 immunotoxin for improved depletion of CCR4+ Treg in nonhuman primates
Source: Mol Oncol. 2018 Jul 3;12(8):1374–82. doi: 10.1002/1878-0261.12331 (PMC6068354; doi:10.1002/1878-0261.12331)
Supplement: Supplementary file 1 — Fig. S1. Monkey CCR4+ Treg depletion (absolute count curves) for M11016 and M11216 in the peripheral blood using the CCR4 immunotoxin. Fig. S2. Monkey CCR4+ Treg depletion for M10916 and M11116 in the peripheral blood using the CCR4 immunotoxin. Fig. S3. Monkey CCR4+ Treg depletion for M10916 and M11116 in the lymph node using the CCR4 immunotoxin. [file MOL2-12-1374-s001.pdf]

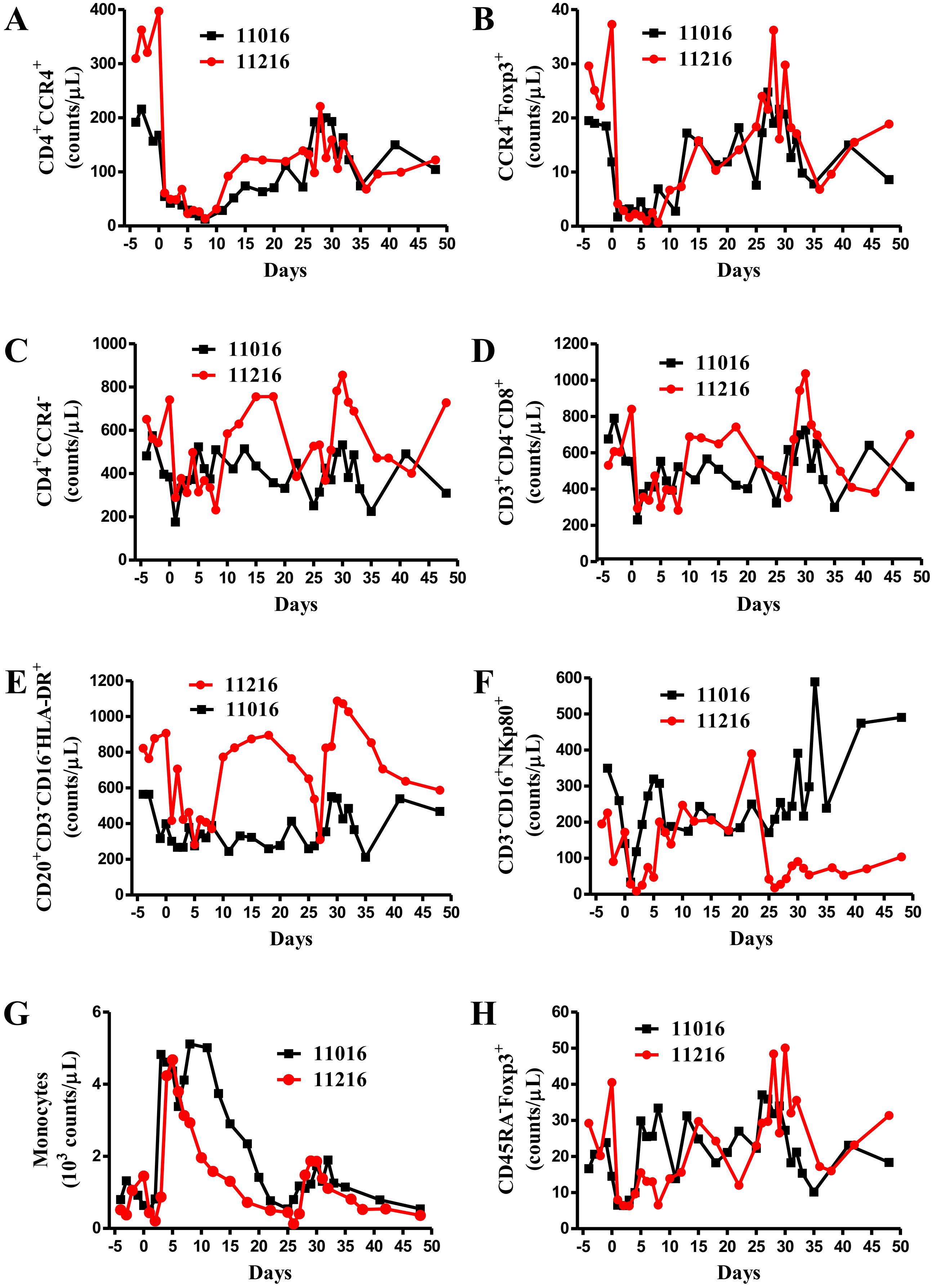
A
CD4+CCR4+ (counts/µL)
11016
11216
Days
B
CCR4+Foxp3+ (counts/µL)
11016
11216
Days
C
CD4+CCR4- (counts/µL)
11016
11216
Days
D
CD3+CD4-CD8+ (counts/µL)
11016
11216
Days
E
CD20+CD3-CD16-HLA-DR+ (counts/µL)
11216
11016
Days
F
CD3-CD16+NKp80+ (counts/µL)
11016
11216
Days
G
Monocytes (10³ counts/µL)
11016
11216
Days
H
CD45RA-Foxp3+ (counts/µL)
11016
11216
Days

Fig. S1. Monkey $CCR4^+$ Treg depletion (absolute count curves) for M11016 and M11216 in the peripheral blood using the CCR4 immunotoxin. The absolute counts were calculated based on the percentage data by flow cytometry analysis and complete blood count analysis. **A)** $CCR4^+$ cell depletion in the peripheral blood was monitored by flow cytometry using the antibodies against human CD4 and CCR4 ($CD4^+CCR4^+$). **B)** $CCR4^+$ Treg depletion in the peripheral blood was monitored by flow cytometry using the antibodies against human CCR4 and Foxp3 ($CCR4^+Foxp3^+$ among the gated $CD4^+$ cells). **C)** The other $CD4^+$ cells in the peripheral blood was monitored by flow cytometry using antibodies against human CD4 and CCR4 ($CD4^+CCR4^-$). **D)** The $CD8^+$ T cells in the peripheral blood were monitored by flow cytometry using the antibodies against human CD3, CD4 and CD8 ($CD3^+CD4^-CD8^+$). **E)** The B cells in the peripheral blood were monitored by flow cytometry using antibodies against human CD20, CD3, CD16 and HLA-DR ($CD20^+CD3^-CD16^-HLA\text{-}DR^+$). **F)** The NK cells in the peripheral blood were monitored using antibodies against human CD3, CD16 and NKp80 ($CD3^-CD16^+NKp80^+$). **G)** Monocytes in the peripheral blood were monitored by flow cytometry using antibodies against human CD14, CD16 and CD11b ($CD14^+CD11b^+$ or $CD14^+CD16^+$, PBMC gating). **H)** The effector Tregs in the peripheral blood were monitored by flow cytometry using antibodies against CD45RA and Foxp3 ($CD45RA^-Foxp3^+$ among the gated $CD4^+$ cells).

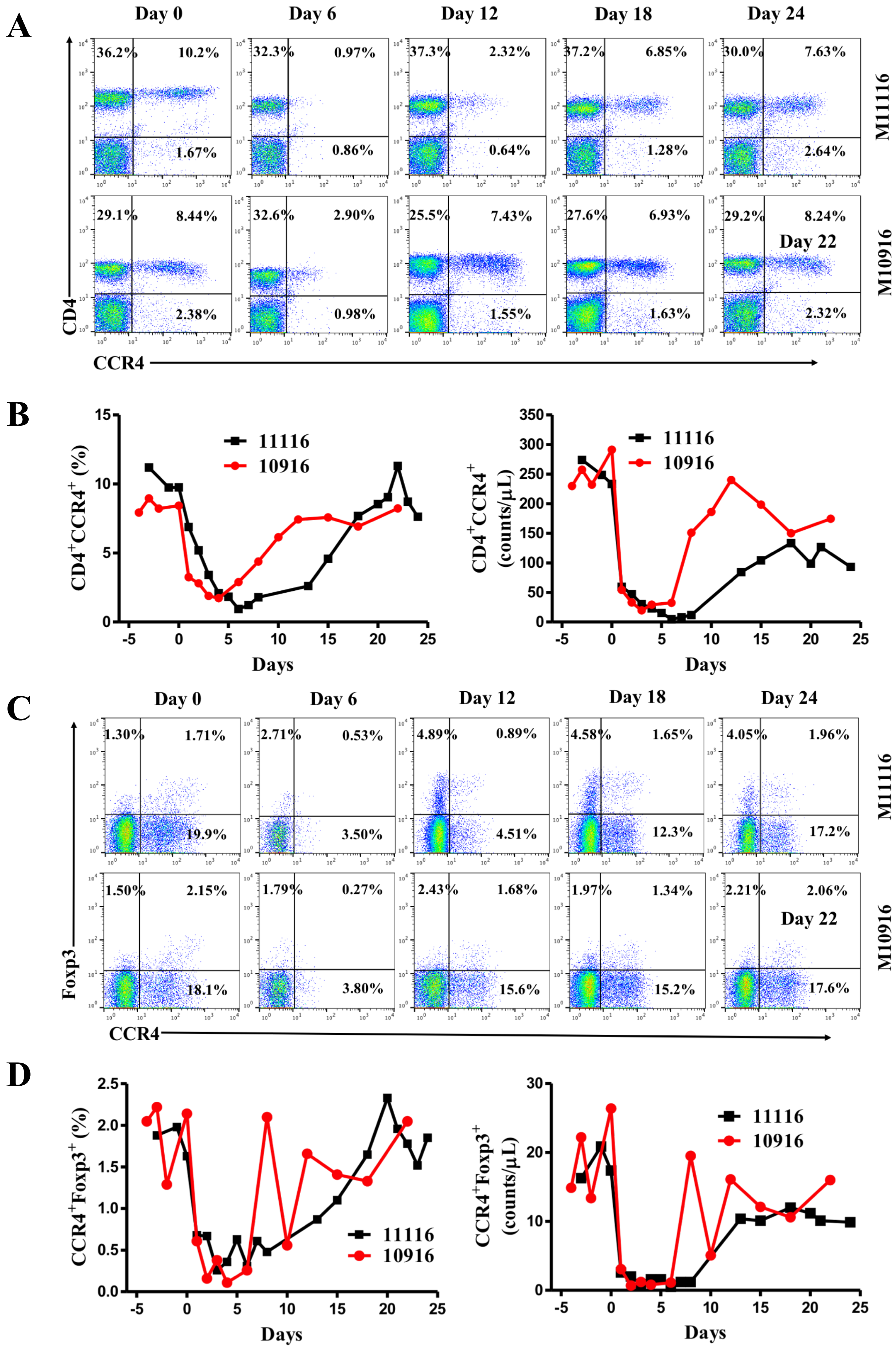
A
Day 0
Day 6
Day 12
Day 18
Day 24
36.2%
10.2%
1.67%
32.3%
0.97%
0.86%
37.3%
2.32%
0.64%
37.2%
6.85%
1.28%
30.0%
7.63%
2.64%
M11116
29.1%
8.44%
2.38%
32.6%
2.90%
0.98%
25.5%
7.43%
1.55%
27.6%
6.93%
1.63%
29.2%
8.24%
Day 22
2.32%
M10916
CD4
CCR4
B
CD4+CCR4+ (%)
11116
10916
Days
CD4+CCR4+ (counts/μL)
C
Day 0
Day 6
Day 12
Day 18
Day 24
1.30%
1.71%
19.9%
2.71%
0.53%
3.50%
4.89%
0.89%
4.51%
4.58%
1.65%
12.3%
4.05%
1.96%
17.2%
M11116
1.50%
2.15%
18.1%
1.79%
0.27%
3.80%
2.43%
1.68%
15.6%
1.97%
1.34%
15.2%
2.21%
2.06%
Day 22
17.6%
M10916
Foxp3
CCR4
D
CCR4+Foxp3+ (%)
CCR4+Foxp3+ (counts/μL)
Days

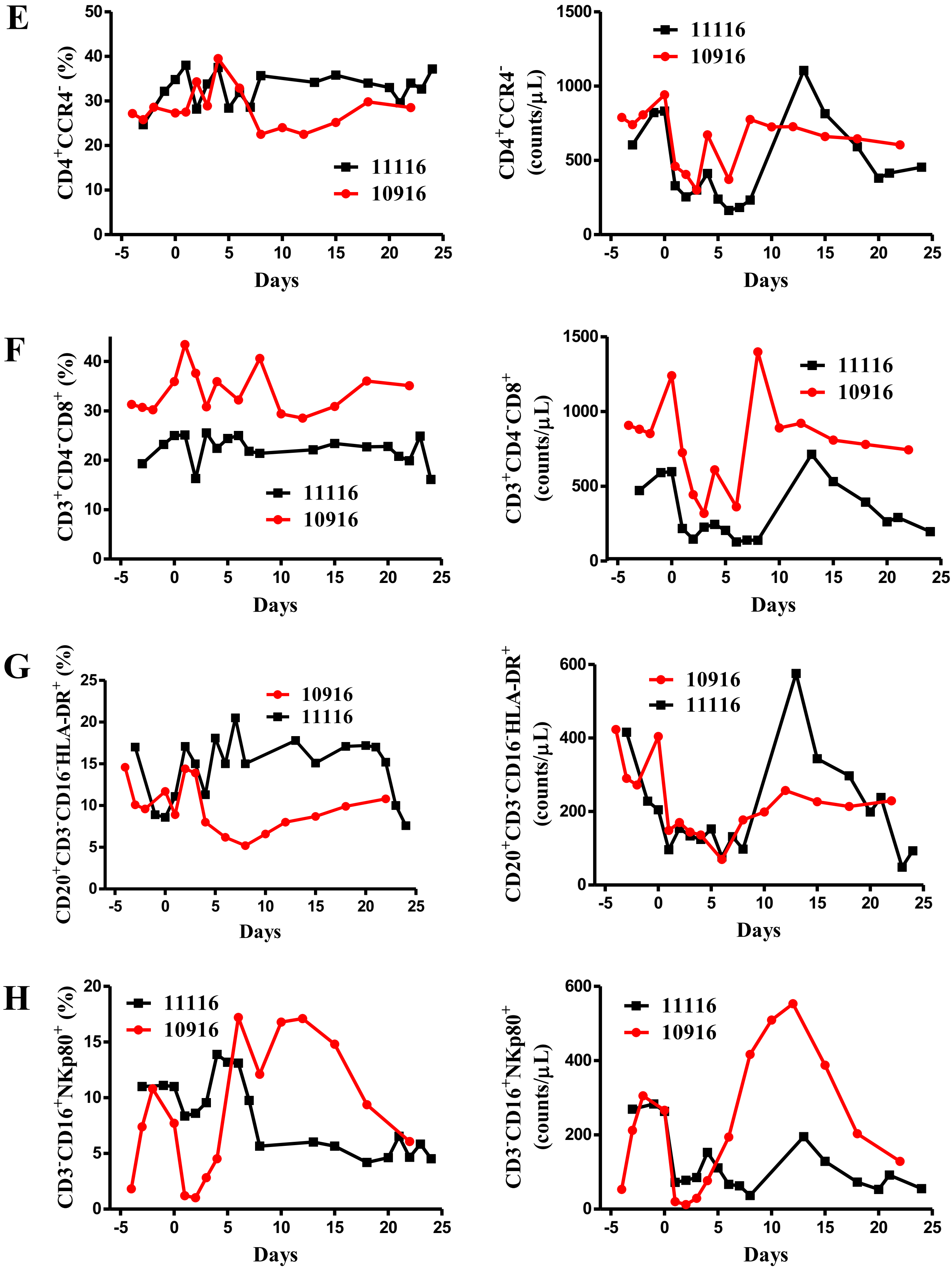

E
CD4+CCR4- (%)
11116
10916
Days
CD4+CCR4- (counts/µL)
F
CD3+CD4-CD8+ (%)
CD3+CD4-CD8+ (counts/µL)
G
CD20+CD3-CD16-HLA-DR+ (%)
CD20+CD3-CD16-HLA-DR+ (counts/µL)
H
CD3-CD16+NKp80+ (%)
CD3-CD16+NKp80+ (counts/µL)

I

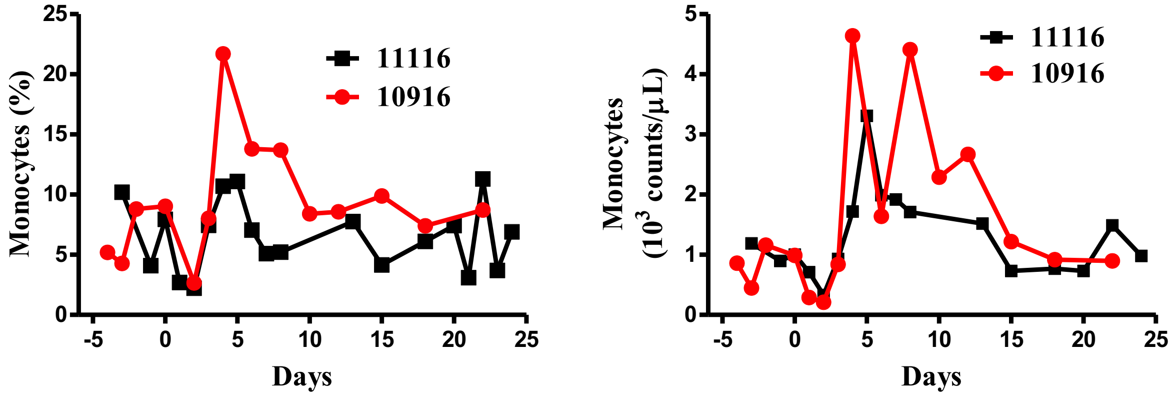
Monocytes (%)
Days
11116
10916
Monocytes
(10^3 counts/µL)
Days
11116
10916

J

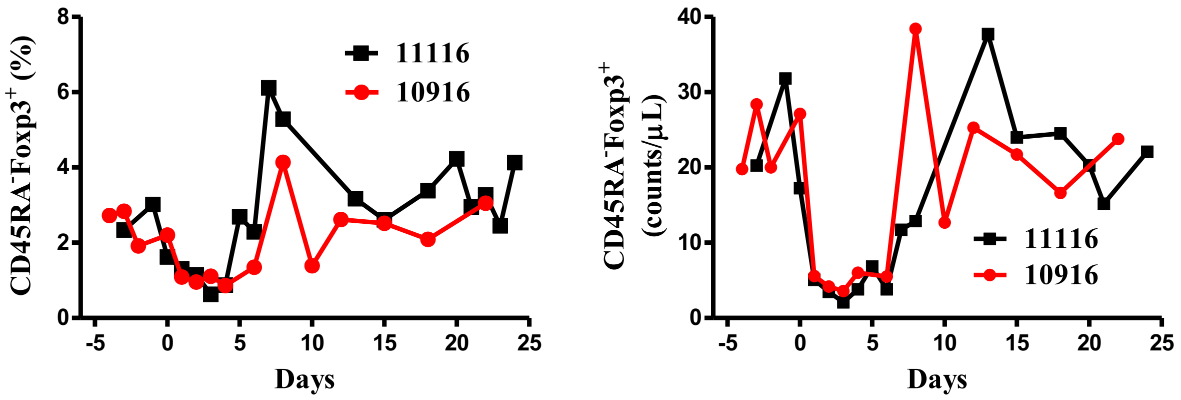
CD45RA$^-$Foxp3$^+$ (%)
Days
11116
10916
CD45RA$^-$Foxp3$^+$
(counts/µL)
Days
11116
10916

K

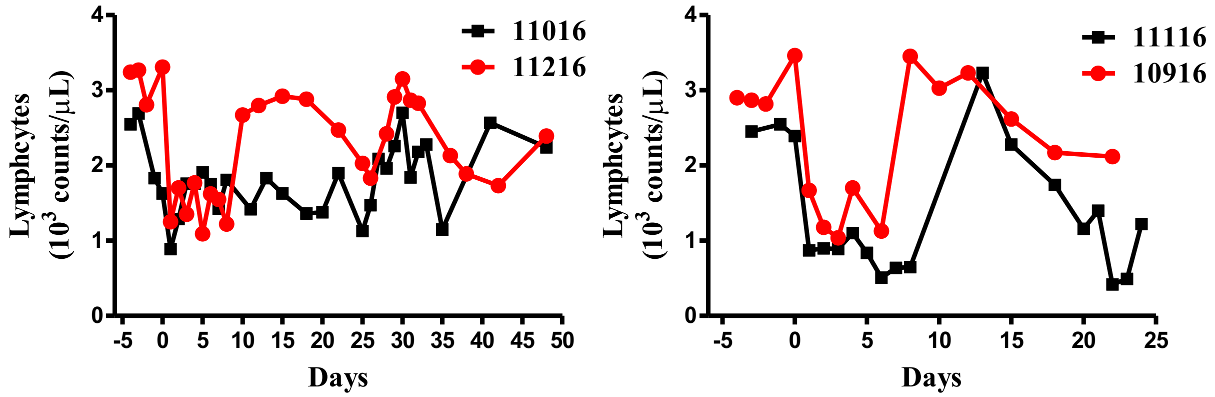
Lymphcytes
(10^3 counts/µL)
Days
11016
11216
Lymphcytes
(10^3 counts/µL)
Days
11116
10916

Fig. S2. Monkey $CCR4^+$ Treg depletion for M10916 and M11116 in the peripheral blood using the CCR4 immunotoxin. The absolute counts were calculated based on the percentage data by flow cytometry analysis and complete blood count analysis. Left panel (2B and 2D-J): percentage value curve; right panel (2B and 2D-J): absolute number curve. **A)** Representative flow cytometry analysis of the $CCR4^+$ cell depletion in the peripheral blood using the antibodies against human CD4 and CCR4. **B)** $CCR4^+$ cell depletion in the peripheral blood was monitored by flow cytometry using the antibodies against human CD4 and CCR4 ($CD4^+CCR4^+$). **C)** Representative flow cytometry analysis of the $CCR4^+Foxp3^+$ Treg depletion in the peripheral blood using the antibodies against human CCR4 and Foxp3 ($CCR4^+Foxp3^+$ among the gated $CD4^+$ cells). **D)** $CCR4^+$ Treg depletion in the peripheral blood was monitored by flow cytometry using the antibodies against human CCR4 and Foxp3 ($CCR4^+Foxp3^+$ among the gated $CD4^+$ cells). **E)** The other $CD4^+$ cells in the peripheral blood was monitored by flow cytometry using antibodies against human CD4 and CCR4 ($CD4^+CCR4^-$). **F)** The $CD8^+$ T cells in the peripheral blood were monitored by flow cytometry using the antibodies against human CD3, CD4 and CD8 ($CD3^+CD4^-CD8^+$). **G)** The B cells in the peripheral blood were monitored by flow cytometry using antibodies against human CD20, CD3, CD16 and HLA-DR ($CD20^+CD3^-CD16^-$ $HLA\text{-}DR^+$). **H)** The NK cells in the peripheral blood were monitored using antibodies against human CD3, CD16 and NKp80 ($CD3^-CD16^+NKp80^+$). **I)** Monocytes in the peripheral blood were monitored by flow cytometry using antibodies against human CD14, CD16 and CD11b ($CD14^+CD11b^+$ or $CD14^+CD16^+$, PBMC gating). **J)** The effector Tregs in the peripheral blood were monitored by flow cytometry using antibodies against CD45RA and Foxp3 ($CD45RA^-$ $Foxp3^+$ among the gated $CD4^+$ cells). **K)** The entire lymphocyte was monitored by complete blood count analysis using HESKA Veterinary Hematology System. Of note, due to the bleeding

event (technical reason, not related to the immunotoxin treatment) with animal M11116 following the scheduled lymph node biopsy on day 8, poor body condition and disordered blood profile were observed which might also affected the flow cytometry analysis data for this particular animal.

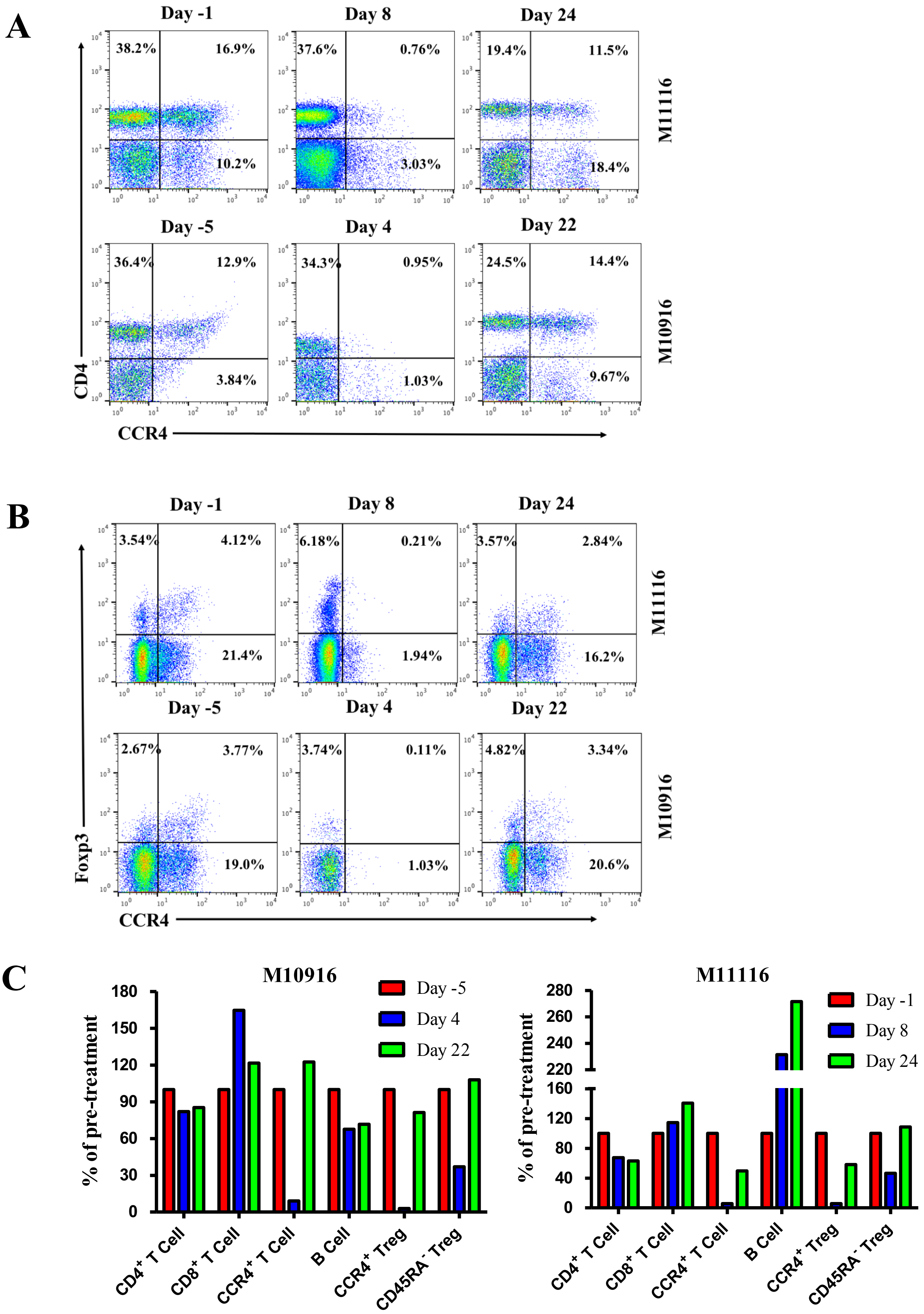
A
Day -1
Day 8
Day 24
38.2%
16.9%
10.2%
37.6%
0.76%
3.03%
19.4%
11.5%
18.4%
M11116
Day -5
Day 4
Day 22
36.4%
12.9%
3.84%
34.3%
0.95%
1.03%
24.5%
14.4%
9.67%
M10916
CD4
CCR4
B
Day -1
Day 8
Day 24
3.54%
4.12%
21.4%
6.18%
0.21%
1.94%
3.57%
2.84%
16.2%
M11116
Day -5
Day 4
Day 22
2.67%
3.77%
19.0%
3.74%
0.11%
1.03%
4.82%
3.34%
20.6%
M10916
Foxp3
CCR4
C
M10916
Day -5
Day 4
Day 22
% of pre-treatment
CD4+ T Cell
CD8+ T Cell
CCR4+ T Cell
B Cell
CCR4+ Treg
CD45RA- Treg
M11116
Day -1
Day 8
Day 24
% of pre-treatment
CD4+ T Cell
CD8+ T Cell
CCR4+ T Cell
B Cell
CCR4+ Treg
CD45RA- Treg

Fig. S3. Monkey $CCR4^+$ Treg depletion for M10916 and M11116 in the lymph node using the CCR4 immunotoxin. The lymph node biopsies were performed before and after each course of the immunotoxin treatment as well as in the end of the study. **A)** Flow cytometry analysis of the lymph node biopsy samples using antibodies against human CD4 and CCR4. **B)** Flow cytometry analysis of the lymph node biopsy samples using antibodies against human CCR4 and Foxp3 ($CCR4^+Foxp3^+$ among the gated $CD4^+$ cells). **C)** Lymph node $CCR4^+$ Treg depletion was monitored by flow cytometry ($CCR4^+$ cells: $CD4^+CCR4^+$, $CCR4^+$ Tregs: $CCR4^+Foxp3^+$ among the gated $CD4^+$ cells, effector Tregs: $CD45RA^-Foxp3^+$ among the gated $CD4^+$ cells). Other cell populations in the lymph node were also monitored by flow cytometry (other $CD4^+$ cells: $CD4^+CCR4^-$; $CD8^+$ cells: $CD8^+CD3^+CD4^-$; B cells: $CD20^+CD3^-CD16^-HLA\text{-}DR^+$).
